# Outcomes of Scarf and Akin Osteotomy with Intra-Articular Stepwise Lateral Soft Tissue Release for Correcting Hallux Valgus Deformity in Rheumatoid Arthritis

**DOI:** 10.3390/ijerph182010667

**Published:** 2021-10-12

**Authors:** Takumi Matsumoto, Yuji Maenohara, Song Ho Chang, Kumiko Ono, Yasunori Omata, Jun Hirose, Sakae Tanaka

**Affiliations:** 1 Department of Orthopaedic Surgery, Faculty of Medicine, The University of Tokyo, Bunkyo-ku, Tokyo 113-8655, Japan; MAENOHARAY-ORT@h.u-tokyo.ac.jp (Y.M.); songhochng@gmail.com (S.H.C.); omatay-ort@h.u-tokyo.ac.jp (Y.O.); tanakas-ort@h.u-tokyo.ac.jp (S.T.); 2 Department of Joint Surgery, Research Hospital, The Institute of Medical Science, The University of Tokyo, Minato-ku, Tokyo 108-8639, Japan; onok-ort@ims.u-tokyo.ac.jp; 3 JCHO Tokyo Shinjuku Medical Center, Department of Orthopaedic Surgery, Shinjuku-ku, Tokyo 162-8543, Japan; j.hirose513@gmail.com

**Keywords:** akin osteotomy, hallux valgus, intra-articular release, joint-preserving arthroplasty, lateral soft tissue release, metatarsosesamoid suspensory ligament, rheumatoid arthritis, scarf osteotomy

## Abstract

Background. The effectiveness of scarf and Akin osteotomy with intra-articular lateral soft tissue release for the correction of hallux valgus (HV) in patients with rheumatoid arthritis (RA) has not been elucidated. Methods. A total of 36 feet in 28 patients with RA who had scarf and Akin osteotomy with intra-articular stepwise lateral soft tissue release between 2015 and 2020 at a single institute were investigated retrospectively, with a mean follow-up period of 32.0 ± 16.9 months. Radiographic evaluations including the HV angle, intermetatarsal angle, and sesamoid position were performed preoperatively and postoperatively. Clinical outcomes were assessed using the Japanese Society of Surgery of the Foot (JSSF) hallux scale and self-administered foot evaluation questionnaire (SAFE-Q). Results. The procedure resulted in significant HV correction, with a recurrence rate of 13.9%. The JSSF scale and all five SAFE-Q subscale scores significantly improved (*p* < 0.05), with no major complications. More than 90% of cases achieved adequate lateral soft tissue release without sacrificing the adductor tendon of the hallux. Conclusions. Intra-articular stepwise lateral soft tissue release in combination with scarf and Akin osteotomy provided satisfactory radiographic and patient-reported outcomes for the correction of HV in patients with RA with minimum lateral soft tissue release.

## 1. Introduction

The feet are essential for a healthy life in humans, and foot problems have been linked not only to pain, difficulties in finding properly fitted footwear, and loss of balance, with an increased risk of falls, but also to general health [1]. Foot and ankle involvement in patients with rheumatoid arthritis (RA) increases with the duration of the disease process and was reported to severely affect quality of life in RA patients [2]. In the past 20 years, significant advancements in pharmacological therapy and treat-to-target strategies aiming at tight control of disease activity have dramatically ameliorated the management of RA, which in turn has changed the trends of orthopedic surgeries in patients with RA; this is evidenced by a decrease in the number of total joint arthroplasties in the large joints [3,4]. In contrast, there is a continuous demand for foot surgeries in patients with RA, probably because of the high prevalence of the affected foot regions from an early stage of the disease and the changes in patients’ expectation for surgical outcome from the achievement of basic activities of daily living to the improvement of their quality of life [3,4]. Resection arthroplasty was pioneered by Hoffmann in 1912; since then, it has been modified by a number of surgeons and widely used as a procedure for correcting forefoot deformity in patients with RA [5]. Although resection arthroplasty leads to satisfactory results in general [6], the fear of insecurity due to the loss of push-off force applied by the forefoot is one of the practical disadvantages of this procedure [7]. Therefore, resection arthroplasty is not considered suitable for patients who need to perform high-level activities of daily living. Owing to recent advances in pharmacological treatment, there has been a trend toward joint-preserving arthroplasty instead of resection arthroplasty for maintaining joint function [8]. Using plantar pressure measurement, a few studies have demonstrated that joint-preserving arthroplasty for rheumatoid forefoot could improve push-off by the forefoot during gait [7,9].

Scarf osteotomy, a horizontal osteotomy of the first metatarsal, is a well-established and one of the most commonly used surgical methods for correcting a hallux valgus (HV) deformity in the non-arthritic joints [10]; however, there are a limited number of studies reporting the management of patients with RA using scarf osteotomy [11,12,13,14,15]. Recently, intra-articular lateral soft tissue release through a medial incision has emerged as an alternative to conventional lateral release through a dorsal incision; however, to the best of our knowledge, there has been no literature reporting its outcome in patients with RA, and it remains a debated topic in the field of HV surgery. The aim of this study was to investigate the clinical and radiographic outcomes of scarf and Akin osteotomy with intra-articular lateral release for correcting HV deformity in RA and to identify the factors related to the recurrence of the deformity.

## 2. Patients and Methods

### 2.1. Patients

This was a retrospective study of 36 feet in 28 consecutive patients with RA who underwent scarf osteotomy for the correction of symptomatic HV deformity between January 2015 and January 2020 at the University of Tokyo Hospital. The cases who could be followed up over 12 months were enrolled in this study. The evaluations at the most recent follow-up as at February 2021 were used for the analysis of outcomes. Based on the results of a previous study by Yano et al. reporting the improvement of the self-administered foot evaluation questionnaire (SAFE-Q) score by joint-preserving forefoot arthroplasty in patients with RA [16], a sample size estimation for detecting a significant difference in the SAFE-Q score by operative procedures was done, assuming a standard deviation of 20 and a difference of 20, with a desired power of 0.80 and an α level of 0.05. The required sample size was estimated as 34, and 36 feet in the present study was considered enough to research the clinical outcomes. All procedures were performed by a single surgeon (T.M.) or by attending surgeons under the supervision of T.M. Indication criteria were symptomatic HV that failed to respond to conservative treatments, including insoles and footwear modification. Contraindications to joint-preserving procedures were severe arthritic changes in the metatarsophalangeal (MTP) joint of Larsen grade IV or more, active rheumatoid synovitis of the forefoot, high RA disease activity, midfoot or hindfoot involvement, or severe instability of the first tarsometatarsal joint. The HV angle (HVA) and intermetatarsal angle (IMA) were not considered as the indication or contraindication criteria. The study was approved by the ethical committees of the University of Tokyo (No. 2674). Written informed consent was obtained from all participants.

### 2.2. Radiographic and Clinical Assessments

Radiographic parameters, including HVA, IMA, and sesamoid position, were measured from the weight-bearing anteroposterior radiographs of the foot taken preoperatively, 3 months postoperatively, 6 months postoperatively, and at the latest follow-up. Non-weight-bearing anteroposterior radiographs of the foot taken 1 month postoperatively were also used to measure radiographic parameters. Sesamoid position was classified as grade 0, 1, 2, or 3 according to the position of the medial sesamoid in relation to the longitudinal line bisecting the first metatarsal shaft [17]. The time to bone union was investigated using a series of postoperative follow-up radiographs. Bone union was defined as the bridging of two or more cortices on the anteroposterior and oblique radiographs of the foot. Delayed union and nonunion were defined as the failure to achieve bone union at 6 months and 12 months after surgery, respectively. Recurrent HV deformity and iatrogenic hallux varus were defined as an HVA of >20° and <0° at the latest follow-up, respectively. To remove the influence of interobserver variability, all radiographic measurements were performed by an independent board-certified member of the Japanese Orthopedic Association who was not otherwise involved in the present study.

Clinical outcomes were assessed preoperatively and at the latest follow-up using the Japanese Society of Surgery of the Foot (JSSF) hallux scale and self-administered foot evaluation questionnaire (SAFE-Q) [18,19,20]. The JSSF hallux scale ranges from 0 to 100 points, with zero identifying the worst status and 100 an optimal healthy status; it includes three major items, such as pain (40 points) and function (45 points), which is further segmentalized into activity limitation, footwear requirement, MTP joint motion, IP joint motion, MTP-IP stability, and callus, and alignment (15 points) [18,19]. The SAFE-Q is an ankle-specific subjective evaluation method by the JSSF that consists of six subcategories (i.e., pain and pain-related, physical functioning and daily living, social functioning, shoe-related, general health and well-being, and sports activity (optional)). Each dimension ranges from 0 to 100, with zero identifying the worst status and 100 an optimal healthy status. Good reliability, validity, and responsiveness have been verified in the JSSF scale and SAFE-Q [18,19,20]. These scores for clinical outcomes were obtained preoperatively and every other year after the surgery. The 28-joint disease activity score based on the erythrocyte sedimentation rate (DAS28-ESR) was used to evaluate disease activity according to the preoperative status [21]. Postoperative complications, including surgical site infection, delayed wound healing, avascular necrosis of the metatarsal head, and reoperation, were investigated using the medical charts.

### 2.3. Surgical Technique

**Figure 1:**
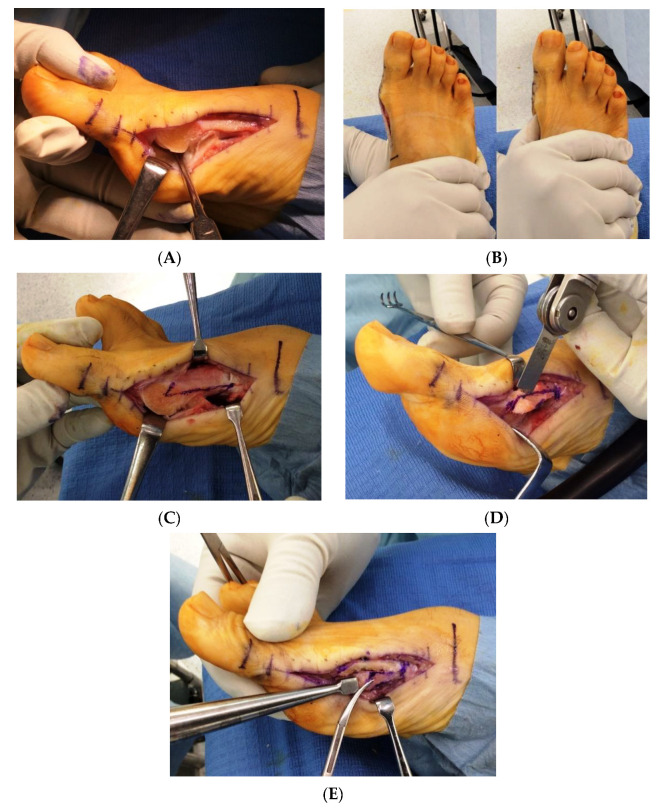
Intraoperative pictures showing the key steps in our procedure for correcting hallux valgus in patients with rheumatoid arthritis. (**A**) Lateral soft tissue release was performed intra-articularly through a single long medial longitudinal skin incision and a capsular incision at the first metatarsophalangeal joint. Blunt dissection of the lateral metatarsosesamoid suspensory ligament was performed using elevatorium followed by sharp dissection using dissection scissors. (**B**) Lateral soft tissue release was performed in a stepwise fashion until the manual correction of the intermetatarsal angle between the first and second metatarsals spontaneously corrected the hallux valgus deformity. It involved the following three steps: (1) dissection of the lateral metatarsosesamoid suspensory ligament, (2) lateral capsulotomy at the joint level, and (3) tenotomy of the adductor tendon insertion into the proximal phalanx. (**C**) Transverse part of scarf osteotomy was performed connecting two apexes; one was located at the center of the metatarsal head and the other at the plantar one-third of the metatarsal 10 mm distal to the first tarsometatarsal joint. (**D**) After the lateral translation of the distal fragment and internal fixations of the proximal and distal fragments with two cannulated headless screws, the prominent medial bone of the proximal fragment was excised in the shape of a wedge. (**E**) Excised fragment was impacted intramedullary in the plantar and proximal regions with the wedge turned backward and upside down.

All surgical procedures were performed in the supine position under spinal anesthesia or local anesthesia using popliteal sciatic nerve block with a thigh tourniquet. A single long longitudinal skin incision was made on the medial side of the first MTP joint from the base of the proximal phalanx to the proximal shaft of the first metatarsal. A capsular incision was then created along the line of the skin incision. After the medial eminence was resected using an oscillating saw, lateral soft tissue release was performed intra-articularly (Figure 1A). Lateral release was performed in a stepwise fashion until the manual correction of the intermetatarsal angle between the first and second metatarsals spontaneously corrected the HV deformity. It involved the following three steps: (1) dissection of the lateral metatarsosesamoid suspensory ligament, (2) lateral capsulotomy at the joint level, and (3) tenotomy of the adductor tendon insertion into the proximal phalanx (Figure 1B). Scarf osteotomy was performed on the first metatarsal with the distal apex at the center of the metatarsal head and the proximal apex at the plantar one-third of the metatarsal; the osteotomy site in the scarf osteotomy was located at 10 mm distal to the first tarsometatarsal joint (Figure 1C). After the osteotomy was completed, the proximal fragment was pulled medially, and the distal fragment was shifted laterally. Then, both the fragments were internally fixed with two cannulated headless screws. The prominent medial bone of the proximal fragment was excised in the shape of a wedge (Figure 1D) and was then impacted intramedullary in the plantar and proximal regions with the wedge turned backward and upside down (Figure 1E) [22]. If a hallux interphalangeus, characterized by a valgus angulation between the distal articular surface and the proximal articular surface of the basal phalanx, was noted fluoroscopically, Akin osteotomy was performed additionally to align the distal and proximal articular surfaces parallel to each other. Akin osteotomy was fixed with a staple or suture [23]. A capsulorrhaphy was performed to correct the plantar dislocation of the abductor hallucis tendon, which is commonly observed in HV deformity, using the pants-over-vest suture fixation technique.

In addition to the above procedures for correcting HV deformity, other procedures for correcting lesser toe deformities, including oblique osteotomy for shortening of the metatarsal, proximal interphalangeal joint arthrodesis, Weil osteotomy, shortening osteotomy of the proximal phalanx, proximal base metatarsal osteotomy for metatarsus adductus, and medial translation of the metatarsal head for the fifth toe, were performed concurrently. After these procedures, with the exception of the Weil osteotomy, a 1.5 mm Kirschner wire was inserted from the toe tip, across the MTP joint and osteotomy site, and into the shaft of the corresponding metatarsals or distal tarsal bones as a transient fixation.

### 2.4. Postoperative Care

Ambulation with weight on the heel in a postoperative shoe was permitted on postoperative day 1. Patients were instructed to perform a manual traction exercise 10 times every hour during the daytime from 1 week to 3 weeks postoperatively by pulling the hallux along the long axis with one hand while stabilizing the first metatarsal with the other hand for 4 s in order to prevent scar tissue adhesion. The frequency of exercise was gradually decreased, but the exercise was continued until the patient could actively perform the toe-spread-out exercise. After the K-wire was removed from the lesser toes at 3 weeks postoperatively, foot-flat gait was permitted. Normal ambulation was allowed after bone union was achieved at all osteotomy sites.

### 2.5. Statistical Analysis

All continuous variables are presented as mean ± standard deviation. The Wilcoxon signed-rank test was used to compare preoperative and postoperative findings. Mann–Whitney’s U-test was used to compare the cases with and without recurrent HV. Receiver operator curve (ROC) analysis was performed by calculating the area under the curve (AUC) to determine the ability of each radiographic parameter to predict recurrent HV. The cutoff value of each radiographic parameter was calculated using the Youden index. For each statistical analysis, a *p* value <0.05 was considered statistically significant. All statistical analyses were performed using JMP Pro version 13.2 (SAS Institute Inc., Cary, NC, USA).

## 3. Results

A total of 36 feet in 28 patients were enrolled in this study; no cases were excluded. The study cohort comprised 1 male and 27 female patients with a mean age of 64.8 ± 9.2 years (range, 45–80 years) at the time of surgery and mean disease duration of 18.5 ± 12.0 years. The mean duration of follow-up was 32.0 ± 16.9 months. Of 28 patients, eight underwent bilateral procedures; four patients underwent bilateral procedures at the same time, and four patients underwent bilateral procedures separately. Out of 28 patients, 8 patients (12 feet), 15 patients (17 feet), and 5 patients (7 feet) were treated with biological disease-modifying anti-rheumatic drugs (bDMARDs), methotrexate, and conventional synthetic disease-modifying anti-rheumatic drugs other than methotrexate, respectively. Intra-articular lateral soft tissue release involved only the dissection of the lateral metatarsosesamoid suspensory ligament in 16/36 feet, both the dissection of suspensory ligament and lateral capsulotomy in 17/36 feet, and all three steps of lateral release, including the dissection of the suspensory ligament, lateral capsulotomy, and adductor tenotomy, in 3/36 feet. Akin osteotomy was performed in all cases. Details of the procedures for correcting lesser toe deformities are summarized in Table 1.

The mean HVA, IMA, and medial sesamoid position measured from preoperative and postoperative radiographs are shown in Table 2. Preoperatively, the mean HVA was 45.5 ± 9.6° (range, 23–64°), which significantly improved to 10.3 ± 11.2° (range, −16–40°) at the latest follow-up, with a mean correction of 35.2 ± 12.7° (*p* < 0.0001). Preoperatively, the mean IMA was 17.5 ± 3.7° (range, 9–26°), which significantly improved to 7.0 ± 4.1° (range, 1–16°) at the latest follow-up, with a mean correction of 10.5 ± 4.2° (*p* < 0.0001). The medial sesamoid position changed significantly from a preoperative value of 3.0 (grade 0: 0 feet, grade 1: 0 feet, grade 2: 1 feet, grade 3: 35 feet) to 1.4 (grade 0: 11, grade 1: 8, grade 2: 10, grade 3: 7) at the latest follow-up (*p* < 0.05). A 66-year-old female patient with moderate RA disease activity, the only current smoker in the cohort, had partial wound dehiscence that took 8 weeks to heal with local wound care. There were no cases of avascular necrosis of the hallux metatarsal head. All patients achieved bony union; the average time to union was 10.5 weeks (range, 6 to 21 weeks).

The mean JSSF scores improved significantly, from a preoperative value of 45.1 ± 14.6 to 90.7 ± 10.8 at the latest follow-up (*p* < 0.0001) (Table 3). All SAFE-Q subscale scores at the latest follow-up (pain and pain-related, 67–86 points; physical functioning and daily living, 68–77 points; social functioning, 67–81 points; shoe-related, 40–62 points; and general health and well-being, 62–84 points) showed significant improvements compared with preoperative SAFE-Q subscale scores (Table 3).

At the latest follow-up, there were four patients (11.1%) with hallux varus deformity and five patients (13.9%) with recurrent HV deformity. There was no case of progressive destruction of the first MTP joint or postoperative hallux deformity that required arthrodesis or revision procedures. Patient demographics and radiographic findings were compared between patients with recurrent HV, classified as the ‘recurrent HV group’, and patients without recurrent HV, classified as the ‘control group’ (Table 2). In the recurrent HV group, DAS28-ESR, the dosage of methotrexate and prednisolone, and the percentage of bDMARD usage were higher compared with those in the control group; however, the difference was significant only in the dosage of methotrexate.

The recurrent HV group had a significantly greater preoperative HVA compared with the control group, but there was no significant difference in the IMA between the groups (HVA, 54.2 ± 7.2° vs. 44.0 ± 9.2°; *p* = 0.0257; IMA, 18.8 ± 3.3° vs. 17.3 ± 3.8°; *p* = 0.400). The AUC of the preoperative HVA for predicting recurrent HV was 0.80. At a cutoff value of 50.9°, the preoperative HVA had a sensitivity and specificity of 80% and 81%, respectively. Patients with recurrent HV had a significantly greater postoperative HVA compared with those without recurrent HV. At 1 month postoperatively, patients with recurrent HV already had a significantly greater HVA compared with those without recurrent HV (19.6 ± 6.8° vs. 6.0 ± 4.7°, *p* < 0.0001). At a cutoff value of 13.6°, the 1-month postoperative HVA had a sensitivity and specificity of 100% and 90%, respectively.

## 4. Discussion

To the best of our knowledge, this is the first study to report the clinical outcomes of scarf and Akin osteotomy with intra-articular lateral soft tissue release for the correction of HV deformity in patients with RA. The procedure resulted in satisfactory patient-reported outcome measure (PROM) scores and radiographic outcomes during a mean follow-up period of 32 months.

In this study, the PROM scores in all subscales regarding pain, physical function, social functioning, shoe-related, and general health improved significantly after the procedure for correcting HV. Joint-preserving arthroplasty for correcting rheumatoid forefoot deformities is becoming increasingly popular as it results in better function and satisfaction in patients than resection arthroplasty [8]; however, only a few studies have evaluated the effectiveness of joint-preserving arthroplasty for correcting rheumatoid forefoot deformities using PROM scores. In a retrospective study of 35 feet, Ebina et al. reported that joint-preserving arthroplasty, including modified scarf osteotomy for the hallux and off-set shortening osteotomy for the lesser toes, significantly improved all SAFE-Q subscale scores [24]. Another retrospective study by Yano et al. including 105 feet demonstrated that joint-preserving surgery including proximal rotational closing-wedge osteotomy of the first metatarsal and shortening oblique osteotomies of the lesser toes also significantly improved all SAFE-Q subscale scores [16]. Additionally, Chao et al. reported a significant improvement in the Short Form 36 score postoperatively in a retrospective study of 37 feet that were treated with a joint-preserving procedure of the first MTP joint, including Ludloff, scarf, and chevron osteotomies [14]. The present study contributes to the accumulation of evidence that joint-preserving arthroplasty for correcting rheumatoid forefoot deformity provides satisfactory subjective results in patients with RA.

Scarf osteotomy is one of the most commonly used surgical methods for correcting a non-arthritic HV deformity [10]; however, there are a limited number of studies reporting its use in patients with RA (Table 4) [11,12,13,15]. Three out of four previous studies reporting the outcome of scarf osteotomy in patients with RA adopted the dorsal approach for lateral soft tissue release [11,13,15], and one did not report lateral soft tissue release [12]. Lateral soft tissue release was described in detail in only one study by Kushioka et al., which reported routine procedures including the release of adductor tendon insertion into the proximal phalanx, transverse metatarsal ligament, and capsule between the first metatarsal and the lateral sesamoid [15]. Unlike these previous studies, the present study adopted a stepwise intra-articular approach for lateral soft tissue release. Detailed description of the lateral soft tissue release in the present study will be valuable for further studies in deepening the debate about the role and extent of the lateral soft tissue release.

Because soft tissue imbalance around the MTP joint plays an important role in the pathophysiology of HV, lateral soft tissue release is a key step in HV surgery as well as osteotomies [25]. Two surgical approaches are commonly used for lateral soft tissue release: the dorsal first web-space approach and the medial intra-articular approach [26,27]. Although the dorsal first web-space approach enables a fairly easy lateral soft tissue release and visual assessment, it can be associated with some risks, including cosmetically undesirable incision scars and avascular necrosis of the first metatarsal head due to potential damage to the first dorsal metatarsal artery, which is the primary source of blood supply to the first metatarsal head [26,27]. In contrast, intra-articular lateral soft tissue release through a single medial incision has advantages over that through a dorsal incision as it reduces the risk of avascular necrosis by limiting extensive soft tissue dissection and avoids scarring on the dorsal side of the foot [28,29]. In this study, we did not observe any cases of avascular necrosis. There are concerns regarding the accuracy of intra-articular lateral soft tissue release because some maneuvers, especially the dissection of the lateral metatarsosesamoid suspensory ligament, must be performed blindly in cases with severe dislocation of the sesamoids. However, some cadaveric studies have demonstrated that lateral soft tissue release, including lateral sesamoid ligament, lateral metatarsophalangeal capsule, and adductor hallucis tendon insertion into the proximal phalangeal base, can be successfully conducted through an intra-articular approach, with little injury to neurovascular bundles [29,30,31]. Some clinical studies also demonstrated that the intra-articular approach could achieve the same corrective effects as the dorsal first web-space approach [27,32,33]. The present study was the first to demonstrate the effectiveness of the intra-articular approach clinically in patients with RA.

In addition to the approach used for lateral soft tissue release, the extent of lateral release remains a matter of debate. In 1923, Silver described a distal soft tissue procedure involving the resection of the medial eminence, lateral capsular release, adductor hallucis tendon release, and medial capsular plication with adductor hallucis transposition [34]. Since then, lateral soft tissue release has been modified by a number of surgeons and has been used for HV surgeries in conjunction with metatarsal osteotomy. In particular, the release of the conjoined insertion of the lateral flexor hallucis brevis tendon and the adductor tendon, lateral sesamoid suspensory ligament, phalangeal band insertion, and transverse metatarsal ligament is under debate. In this study, we adopted a stepwise procedure consisting of three steps: dissection of the lateral metatarsosesamoid suspensory ligament, lateral capsulotomy at the joint level, and tenotomy of the adductor tendon insertion into the proximal phalanx. However, the final step was required only in three cases. This stepwise procedure is based on a cadaveric study by Schneider et al. [35]. The study demonstrated that the division of the lateral metatarsosesamoid suspensory ligament was the key step in the successful correction of the HVA, the IMA, and sesamoid subluxation, whereas transection of the lateral collateral ligament led to a limited correction of the HVA and IMA, with no effect on sesamoid subluxation. Schneider et al. also reported that the release of neither the deep transverse metatarsal ligament nor the adductor hallucis had an effect on the HVA and IMA. A clinical study by Augoyard et al. validating sequential lateral soft tissue release for the surgical treatment of HV also demonstrated that the dissection of the metatarsosesamoid suspensory ligament and phalangeal insertional band played a key role in deformity correction, while the release of the adductor from the fibular sesamoid had a limited effect on deformity correction [36]. The present study suggested that sequential lateral soft tissue release beginning with the dissection of the lateral metatarsosesamoid suspensory ligament was effective and did not require the complete release of the abductor hallucis tendon or transverse metatarsal ligament, even in patients with RA, who are likely to have more severe deformities than the general population.

In the present study, the recurrence rate of HV deformities was 13.9%, which was not worse compared with that reported in previous studies on joint-preserving surgery in patients with RA (0–40%) [11,13,14,15,16,37,38]. Reported risk factors for recurrent HV after joint-preserving procedures in patients with RA include severe preoperative deformity, high preoperative RA disease activity, splayfoot, and hindfoot malalignment [13,15,39,40]. Patients with recurrent HV had a higher preoperative HVA compared with those without recurrent HV, suggesting an association between severe preoperative deformity and recurrent HV. The present study also demonstrated that patients with recurrent HV had a significantly greater HVA at 1 month postoperatively compared with those without recurrent HV, suggesting that postoperative HV deformity was largely due to the undercorrection of severe preoperative deformity rather than recurrence after adequate correction. The results of ROC analysis indicate that careful consideration is required for adapting joint-preserving procedures for cases with an HVA of 50° or more. RA disease activity was higher in patients with recurrent HV compared with that in patients without HV, but the difference was not significant. The small sample size and lack of patients with high disease activity undergoing joint-preserving procedures in the present study make it difficult to assess the relationship between disease activity and recurrent HV. We believe that midfoot or hindfoot involvement has a negative effect on the maintenance of forefoot deformity correction, and patients with midfoot or hindfoot involvement were not included in the present study. Therefore, the association of midfoot or hindfoot involvement with recurrent HV was not analyzed in the present study. Exclusion from the application of joint-preserving procedure was judged by an experienced foot and ankle surgeon but did not follow such a strict guideline as using radiographic parameters in the present study. Although hallux valgus is a complex three-dimensional deformity, most of the commonly used parameters for evaluating hallux valgus, such as the hallux valgus angle, can only explain the deformity in the horizontal plane and might not evaluate the real three-dimensional deformity of the hallux [41,42]. Further studies with consideration of the three-dimensional deformity of the hallux will be needed to expand the application of joint-preserving arthroplasty in rheumatoid forefoot deformity.

Hallux varus is one of the major complications after HV surgery; the prevalence of hallux varus ranges between 2% and 15% after different types of surgeries [12,15,16,43]. In this study, there were four cases (11.1%) of postoperative hallux varus; however, the deformities were not progressive or symptomatic in all these cases, and no case required reoperation at the latest follow-up. All cases of hallux varus deformity had a varus deformity of 10° or less except one. Importantly, one case with an HVA of − 16° was one of the three cases in which all three steps of lateral soft tissue release were performed. Iatrogenic hallux varus can be caused by various factors, including excessive resection of the medial eminence, excision of the fibular sesamoid, excessive lateral soft tissue release, overcorrection of the intermetatarsal angle or HV interphalangeus, excessive medial capsulorrhaphy, and aggressive postoperative bandaging. In the present study, the stepwise intra-articular approach enabling the minimum lateral soft tissue release might have contributed to the low prevalence of severe iatrogenic hallux varus that required revision procedures.

Our study has some limitations. First, it was a retrospective study; however, all patients who underwent the procedure during the study period were enrolled in this study, minimizing the risk of selection bias resulting from its retrospective design. Second, the present study did not include a control group; therefore, the superiority of scarf and Akin osteotomy to resection arthroplasty or other procedures could not be concluded. Third, we adopted several procedures for correcting lesser toe deformities depending on the condition of individual toes; therefore, our PROM scores may not be attributed solely to the procedures for correcting HV. Fourth, we adopted our joint-preserving procedures only for the patients without midfoot or hindfoot involvement. The results of the present study might not be applicable for those with these pathologies. Finally, the follow-up duration in this study was relatively short. Due to the progressive nature of RA, a longer follow-up period may be necessary to validate our findings.

## 5. Conclusions

Scarf and Akin osteotomy, which is one of the established methods for correcting the hallux valgus deformity in the non-arthritic joints, was found to provide satisfactory clinical and radiographic outcomes also in patients with RA. Stepwise intra-articular lateral soft tissue release could achieve adequate lateral release without sacrificing the adductor tendon in more than 90% of cases.

## Figures and Tables

**Table 1:** Procedures for correcting lesser toe deformities.

**Procedures**	**Second Toe**	**Third Toe**	**Fourth Toe**	**Fifth Toe**
Oblique osteotomy for shortening of the metatarsal	32	27	23	11
PIP joint arthrodesis *	12	6	6	0
Shortening osteotomy of the proximal phalanx	3	1	0	0
Abduction osteotomy of the metatarsal	1	1	0	0
Weil osteotomy	0	4	0	0
Medial translation of the metatarsal head	0	0	0	13

PIP, proximal interphalangeal. * In all cases, PIP joint arthrodesis was performed along with oblique osteotomy for shortening of the metatarsal in the same toe.

**Table 2:** Comparisons of patient demographics and radiographic findings between cases with and without recurrent hallux valgus.

Items		Total (*n* = 36)	Recurrent Deformity (*n* = 5)	No Recurrent Deformity (*n* = 31)	*p* Value
Age, years		64.8 ± 9.2	64.2 ± 8.2	64.9 ± 0.5	0.8712
Duration of disease, years		18.5 ± 12.0	10.8 ± 5.2	19.7 ± 12.4	0.1266
Follow-up period, months		30.9 ± 16.2	36.2 ± 22.0	30.1 ± 15.4	0.4420
Methotrexate, mg/week		5.1 ± 4.5	9.0 ± 6.0	4.5 ± 4.0	0.0344 *
Prednisolone, mg/day		1.4 ± 2.0	2.6 ± 2.5	1.2 ± 1.9	0.1595
Use of bDMARDs, *n* (%)		12 (33)	2 (40)	10 (32)	0.8053
DAS28-ESR		2.8 ± 0.9	3.2 ± 1.2	2.7 ± 0.9	0.2930
Body mass index, kg/m^2^		20.8 ± 2.7	22.0 ± 2.5	20.7 ± 2.7	0.2956
Radiographic parameters					
Preoperative	HVA, °	45.5 ± 9.6	54.2 ± 7.2	44.0 ± 9.2	0.0257 *
	IMA, °	17.5 ±3.7	18.8 ± 3.3	17.3 ± 3.8	0.4000
	Sesamoid position	0:1:35:0	0:0:0:5	0:0:1:30	>0.999
1 month postoperative	HVA, °	7.9 ±6.8	19.6 ± 6.0	6.0 ± 4.7	<0.0001 *
	IMA, °	6.5 ± 4.3	10.1 ± 4.4	5.9 ± 4.0	0.0383 *
	Sesamoid position	16:13:7:0	0:4:1:0	16:9:6:0	0.0585
3 months postoperative	HVA, °	10.2 ± 6.8	21.0 ± 6.0	8.4 ± 5.1	<0.0001 *
	IMA, °	7.9 ± 3.9	10.1 ± 6.4	7.6 ± 3.4	0.0886
	Sesamoid position	11:17:5:3	0:3:1:1	10:14:4:2	0.4160
6 months postoperative	HVA, °	9.7 ± 7.7	20.1 ± 6.3	8.0 ± 6.6	0.0005 *
	IMA, °	7.1 ± 4.2	9.8 ± 5.2	6.7 ± 3.9	0.1331
	Sesamoid position	11:13:10:2	0:1:4:0	11:12:6:2	0.0418 *
Latest follow-up	HVA, °	10.3 ± 11.2	29.2 ±7.6	7.2 ± 8.4	<0.0001 *
	IMA, °	7.0 ± 4.1	10.0 ±5.2	6.5 ± 3.8	0.0801
	Sesamoid position	11:8:10:7	0:1:2:2	11:7:8:5	0.2036

bDMARDs, biological disease-modifying antirheumatic drugs; DAS28-ESR, 28-joint disease activity score based on erythrocyte sedimentation rate; HVA, hallux valgus angle; IMA, intermetatarsal angle between the 1st and 2nd metatarsals; *, statistically significant between cases with and without recurrent hallux valgus.

**Table 3:** Comparisons of the JSSF and SAFE-Q scores before and after surgeries.

Scores	Preoperative	Latest Follow-Up	*p* Value
JSSF hallux scale score			
Pain (40 points)	22.3 ± 9.1	37.9 ± 4.2	<0.0001 *
Function (45 points)	22.7 ± 7.9	38.9 ± 6.7	<0.0001 *
Alignment (15 points)	0.7 ± 2.8	14.0 ± 2.6	<0.0001 *
Total (100 points)	45.1 ± 14.6	90.7 ± 10.8	<0.0001 *
SAFE-Q score			
Pain and pain-related	67.1 ± 18.8	84.6 ± 15.7	<0.0001 *
Physical functioning and daily living	67.6 ± 22.3	79.1 ± 19.3	0.0053 *
Social functioning	66.8 ± 26.9	84.0 ± 22.7	0.0019 *
Shoe-related	39.7 ± 19.5	61.6 ± 18.0	<0.0001 *
General health and well-being	62.0 ± 27.8	85.5 ± 17.8	<0.0001 *

JSSF, Japanese Society of Surgery of the Foot; SAFE-Q, self-administered foot evaluation questionnaire; *, statistically significant.

**Table 4:** Literature reports on the clinical and radiographic outcomes of scarf osteotomy for correcting hallux valgus deformity in patients with rheumatoid arthritis.

Study	Procedures for Correcting HV	Addition of Akin Osteotomy	No. of Feet	Mean FU Period, Months	Approach for Lateral Release	Extent of Lateral Release	Preoperative HVA, °	HVA at the Latest FU, °	Cases with Recurrent HV, %	Cases with Iatrogenic Hallux Varus, %	Clinical Outcomes
Berg et al. [9]	scarf osteotomy	if necessary, 30%	20	65	dorsal	NR	41	28	40% (no definition of recurrent HV)	0%	Patient satisfaction rate: 79%
Barouk et al. [10]	scarf osteotomy	routine, 100%	55	75	NR	NR	NR	NR	5% (no definition of recurrent HV)	some	none
Bhavikatti et al. [11]	scarf osteotomy	0%	66	51	dorsal	NR	32	14	5% (HVA >18° and IMA >11°)	NR	AOFAS score: 39.8 ⇒ 88.7, Subjective report: excellent 74%, good 13.5%, fair 10.5%; poor 1%
Kushioka et al. [13]	scarf osteotomy with medial capsule interposition	if necessary, 4%	76	35	dorsal	routine fashion: adductor tendon insertion into the PP, DTML, and capsule between the first metatarsal and the lateral sesamoid	50.8	12.8	16% (HVA >20°)	9%	JSSF RA foot and ankle scale score: 52.2 ⇒ 76.9, JSSF hallux scale score: 38.2 ⇒ 74.5, SAFE-Q score: no preoperative data ⇒ 78.8/71.3/70.2/83.1/60.4
Our study	scarf osteotomy	if necessary, 100%	36	32	intra- articular	stepwise fashion: suspensory ligament, lateral capsule, and adductor tendon insertion into the PP	45.5	10.3	14% (HVA >20°)	11%	JSSF hallux scale score: 45.1 ⇒ 90.7, SAFE-Q score: 67.1/67.6/66.8/39.7/62.0 ⇒ 84.6/79.1/84.0/61.6/85.5

FU, follow-up; HVA, hallux valgus angle; HV, hallux valgus; NR, not reported; IMA, intermetatarsal angle between the 1st and 2nd metatarsal; PP, proximal phalanx; DTML, deep transverse metatarsal ligament; AOFAS, American Orthopedic Foot and Ankle Society; JSSF, Japanese Society of Surgery of the Foot; RA, rheumatoid arthritis; SAFE-Q, self-administered foot evaluation questionnaire.

## Data Availability

Data supporting the findings of this study are available on request from the corresponding author (T.M.).
